# Buck–Boost DC–AC converter based on coupled inductors

**DOI:** 10.1038/s41598-024-67086-7

**Published:** 2024-07-30

**Authors:** Dina S. M. Osheba, Belal M. Goda, Awad E. Elsabbe, Ashraf Zein Elden

**Affiliations:** Department of Electrical Engineering, Faculty of Engineering, Menoufia University, Shebin El-Kom, 32511 Egypt

**Keywords:** Coupled inductor, High voltage gain, Single stage conversion system, DC–AC converter, Engineering, Electrical and electronic engineering

## Abstract

In this paper, a single stage buck–boost DC–AC converter based on coupled inductors is presented for renewable energy and electric vehicle applications. The proposed topology works with only three semiconductor switches, two diodes, and three coupled inductors to transfer input DC voltage to a high gain or low gain output AC voltage. A coupled inductor is used instead of normal inductors, which will reduce core and size requirements. The sinusoidal pulse width modulation strategy is used in this paper for controlling the main switch. There are many merits in the presented topology, like high gain up to five times of input voltage, compact size, less number of components, which results in reducing the overall cost, reducing switching loss, and increasing the converter efficiency. The simulation study is carried out using MATLAB/SIMULINK to simulate the operation of the proposed converter. Also, an experimental setup is built up to examine the actual operation of the proposed converter. There is a good agreement between simulation and experimental results which increases the validation and confidence of the model.

## Introduction

The worldwide attention to sustainable energy has grown stronger, placing a greater emphasis on renewable sources as the main contributors to energy needs. Several of these energy systems such as photovoltaics (PV), fuel cells, and UPS systems, naturally produce DC voltage. Effectively transmitting power from DC sources to AC loads or the electrical grid requires an appropriate power conversion system (PCS).

Two-stage conversion systems (TSCSs) are commonly employed in this scenario, utilizing either a boost converter or a high-gain DC–DC converter in conjunction with a DC–AC inverter. This configuration aims to transfer power from a low-input voltage DC source to a high-voltage AC load through two stages. Some of these converters buck or boost the DC input voltage in the first stage, then convert this high DC voltage to AC voltage through a conventional inverter in the second stage^1^. The other converters of this TSCS type of transfer DC input voltage to AC voltage in the first stage, then buck or boost this AC voltage in the second stage^2^. This is a typical two stage PCS. Several two stage PCSs have been reviewed in^3,4^, typically involving a cascade connection of a conventional high-gain DC–DC converter and a DC–AC inverter. Employing a two-stage process for converting DC voltage to AC voltage tends to escalate both size and cost. Additionally, utilizing a high-gain DC–DC converter necessitates a higher count of passive elements to achieve the desired amplification. Nevertheless, this type of power conversion system (PCS) is plagued by drawbacks, encompassing increased dimensions, elevated costs, a higher number of components, diminished efficiency, and compromised reliability. Proposed a TSCS topology called Single-Phase High-gain Bidirectional DC/AC Converter Based on a high step-up/step-down DC/DC Converter and a dual-input DC/AC Converter^5^. This topology has high efficiency reaching to 95% but it suffers from complexity in control because it uses bidirectional DC/AC converter. Another proposed a TSCS topology called Isolated and bidirectional two-stage DC/AC converter with grid-forming virtual inertia and high ripple on the DC bus for Single-Phase grid applications^6^. This topology incorporates a film capacitor in lieu of an electrolytic capacitor on the DC-Bus, resulting in a reduction in volume. Furthermore, this modification contributes to heightened efficiency. The overall drawbacks of TSCSs arise when they function with exceedingly low input voltages. Under such conditions, the boost converter is compelled to operate at exceptionally high duty ratios, leading to increased losses and issues with reverse recovery voltage. Also, using a high gain DC–DC converter results in a greater number of components, large size, high cost, lower reliability, lower efficiency and complexity in control.

Single-stage conversion systems (SSCSs) are formed by merging both DC–DC and DC–AC conversion processes, which in turn results in high efficiency, more reliability, and low cost^7^.

As (SSCS) gives several advantages compared with a two-stage conversion system, research focus has shifted towards SSCS^8–12^. A topology named a boost DC–AC converter is proposed in^8^. In this topology, two traditional boost converters, operating with a 180° phase shift, are employed, one for harnessing the positive half cycle and the other for capturing the negative half cycle. This topology has fewer numbers of components, which results in low cost and simplicity in control. However, this configuration is plagued by issues such as high switching losses, electromagnetic interference (EMI) problems, and limited functionality, as it operates exclusively in boost mode.

Another topology^9^ presented two inductor topologies based on the buck–boost principle. This topology has some merits, like using less number of switches (only four), low switching losses (only two switches out of four operate at high switching frequency), compact size and working in buck–boost mode. However, the primary downside of this configuration is its restricted amplification capability, offering a boost of only approximately 1.5 times the input. Another single-phase single stage converter for PV applications is described in^10^. Although this topology has a few numbers of components that results in low cost, smaller size high efficiency, and limited gain either. A new topology based on the output unfolding circuit is proposed in^11^. The advantages of this topology are that it can work under a wide range of input voltages and has high efficiency, but it suffers from high switching losses, greater number of components and a complex control algorithm. A topology named bidirectional single stage isolated DC–AC converter^12^, has some great advantages like it can work in DC–AC or AC-DC conversion mode and useful in UPS systems. Moreover, a wide range of input/output voltages is obtained, and high-voltage power semiconductor switches operate with soft switching, reducing associated power losses. However, it has some drawbacks like using a larger number of semiconductor switches (8 switches) and also complexity in control which combine PWM and frequency control.

Coupled inductors are used instead of traditional inductors aiming to reduce component count, minimize size, and achieve higher gain. A converters based on coupled inductor are presented in^13,14^, which achieve relatively more gain. However, the leakage inductance problems lead to limited low power applications only.

Normally, when coupled inductors are used in power converters, the leakage inductance of coils causes several undesirable effects, like high voltage stress on switches and energy loss. Several converter topologies that can mitigate the adverse effects of leakage inductance are proposed in^15–24^.

Normally when the coupled inductors are used in power converters, the leakage inductance of coils causes several undesirable effects, like high voltage stress on switches and energy loss. Several converter topologies that can mitigate the adverse effects of leakage inductance are proposed in^15–24^. An innovative high-efficiency, high step-up boost-flyback converter is introduced in^15^. This converter efficiently recovers leakage energy to the output terminal while substantially minimizing switch voltage stress. Leveraging the coupled inductor technique, it achieves a remarkable high step-up voltage gain. However, it is primarily designed for low-power applications and operates as a DC–DC converter. A high step-up converter with a coupled-inductor is presented in^16^. In this topology, the voltage gain can be greatly heightened due to the utilization of a coupled inductor with a lower turns’ ratio. The diode short-circuits and reverse-recovery problems can be solved because all the diodes possess voltage clamped properties. On the other hand, it is more suitable for a DC power conversion mechanism with a high voltage variation range. A new switched-coupled-inductor cell is presented in^17^, the energy of the leakage inductance is transferred to the load in a non-oscillatory way, but it is more suitable for DC applications. Low voltage stress and high efficiency voltage-clamped coupled-inductor boost converter is proposed^18^. This topology can work with high boost applications and works as DC–DC converter. The converter outlined in^20^ stands out due to its advantageous characteristics, including high gain, reduced switching losses, compact size, simplified control and the utilization of a coupled inductor in place of two conventional inductors. This design choice leads to decreased core and space requirements. Therefore, this topology is used as a part of single-stage conversion scheme. The converter presented in^21^ gives relatively more gain, but it suffers from high input current.

A structure for high step-up DC–DC converters based on the combination of A-SL and CI techniques is propounded in^22^. This topology provides high voltage gain upto 13.33 with reduced voltage stress on switches, low output voltage spicks, and working only as DC–DC converter. Other topologies presented in^23,24^ have improved reverse recovery performance and low voltage stresses on switches. Therefore, these topologies are used as a part of single-stage conversion scheme.

A coupled inductor-based boost microinverter featuring dual mode time sharing operation for renewable energy applications is outlined in^25^. This innovative topology significantly improves performance in renewable energy deployment, boasting an impressive 97% efficiency with only 1.1% total harmonic distortion in output. However, challenges arise with high inductor ripple current constraints in continuous mode operation. Moreover, the design's reliance on large inductors reduces power conversion efficiency at higher ratings.

In^26^, a coupled-inductor-Based DC–AC multilevel converter featuring a reduced number of switches is presented. This novel topology introduces a hybrid bidirectional converter tailored for HMG applications, leveraging coupled inductors to mitigate output current ripple and system complexity. Despite achieving a THD of 3.1% in voltages and currents, challenges emerge due to the inclusion of six additional diodes compared to a two-level converter, as well as the absence of commercial modules incorporating all necessary semiconductors for a leg. A high step-up DC–DC converter featuring active switched inductor and coupled inductor is presented in^27^. This topology enhances efficiency, minimizes voltage stresses on power switches, and recycles energy. Notably, it is utilized within a single-stage conversion scheme.

In^28^, a coupled-inductor-based buck–boost AC–DC Converter with balanced DC output voltage is introduced. This paper unveils a pioneering design of an AC–DC converter based on coupled inductors. However, it is noteworthy that this converter utilizes an AC input supply and involves a higher number of switches.

In^29^, a design and analysis of a coupled-inductor switched-capacitor boost DC–AC inverter is outlined. This paper proposes a straightforward structure of a coupled-inductor switched-capacitor inverter (CISCI) aiming for high-gain boost DC–AC conversion and closed-loop regulation. However, it operates as TSCSs and requires a higher number of components.

**Figure 1:**
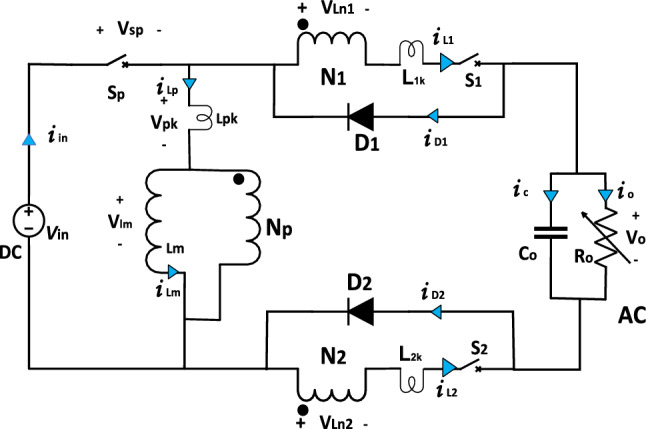
Proposed converter topology.

In this paper, a single stage high gain buck–boost DC–AC converter based on three coupled inductors with only three semiconductor switches is presented. The proposed converter, which is shown in Fig. 1, has the following advantages:

(i) The topology achieves high gain through the incorporation of a coupled inductor, enhancing its performance even under low input voltage conditions.
(ii) Out of the three active switches, only one operates at high frequency, leading to reduce overall switching losses during operation.
(iii) The design utilizes a minimal number of semiconductor switches (only three), resulting in a cost-effective and compact size.
(iv) The operation of the topology is based on simple sinusoidal pulse width modulation (SPWM), eliminating the need for complex modulation techniques.
(v) The topology is versatile, functioning in both buck and boost modes, adding to its flexibility and applicability in different scenarios.

The paper is structured in the following manner: “Introduction” section provides the introduction, “Circuit configuration and modes of operation” section elaborates on the configuration and modes of the proposed converter operations, “Mathematical analysis” section details the mathematical analysis, and presents the design of system components. In “Design of the components” section, simulation and experimental results are discussed, while “Simulation study” and “Experiential study” sections further delve into the presentation of simulation and experimental findings.

## Circuit configuration and modes of operation

The proposed topology as shown in Fig. 1 consists of three active power switches (${S}_{{\varvec{p}}}$, ${S}_{1}$ and ${S}_{2}$), two passive switches (${D}_{1}$ and ${D}_{2}$), an output capacitor (${C}_{0}$) and three coupled inductors (Np, N1, N2). The two switches ${S}_{1}$ and ${S}_{2}$ are IGBTs with antiparallel diode with them. Out of three switches, only one switch ${S}_{{\varvec{p}}}$ operates at high frequency and the remaining switches operate at frequency of output voltage. The antiparallel diodes with switch complete the current loops during the operation. The operation of the proposed topology is the same and symmetrical for the positive and negative halves of the full cycle of the output voltage. The switch ${S}_{{\varvec{p}}}$ operates with SPWM, ensuring the transfer of power from input DC source to output AC load in each cycle. Our analysis will be considered on positive half cycle of full wave and the negative half cycle is the same as the positive half cycle. Our analysis will be considered in steady-state operation.

The following assumptions are made to explain the steady-state operation of the proposed topology. These assumptions are: -

(i) All parasitic components except leakage inductance of the coupled inductor are neglected.
(ii) The on-state resistance of the switches and forward voltage drop of the diodes are ignored.
(iii) Capacitor (Co) is large enough to be considered it as a constant voltage source over a switching cycle.
(iv) The topology is operating under continuous conduction mode.

### Mode 1 (${{\varvec{t}}}_{0},{{\varvec{t}}}_{1}$)

**Figure 2:**
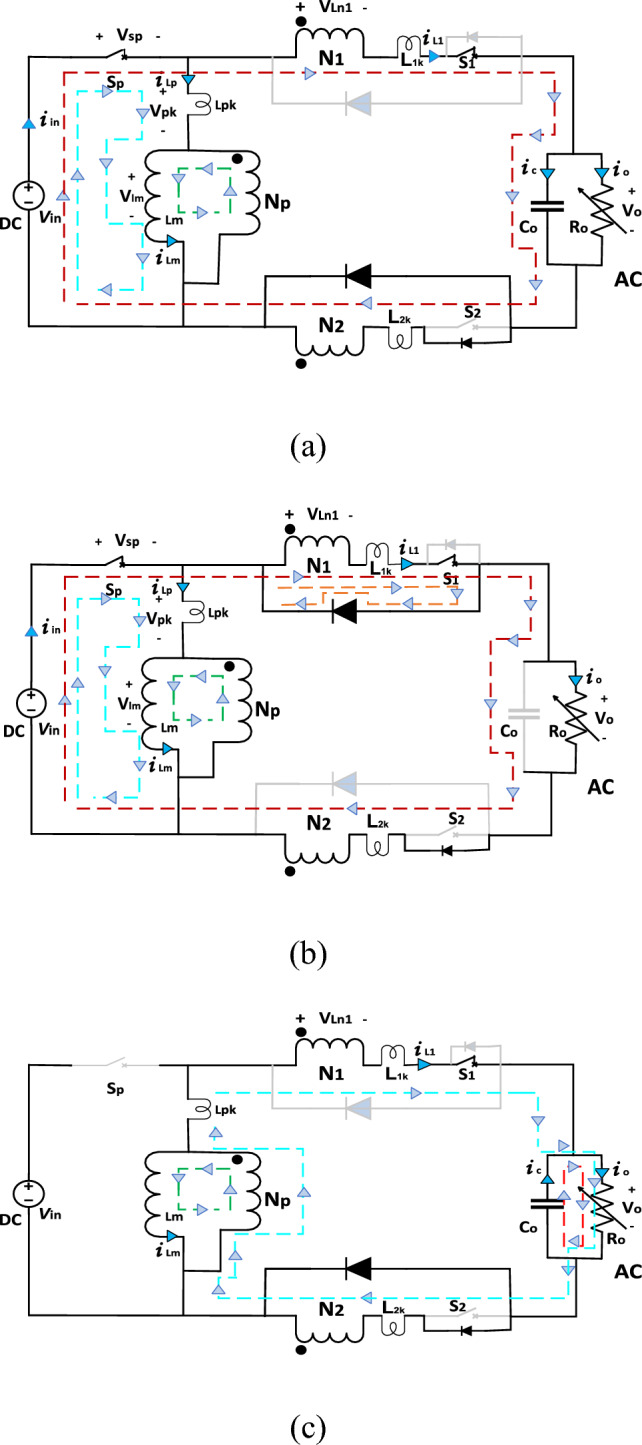
Modes of operation.

**Figure 3:**
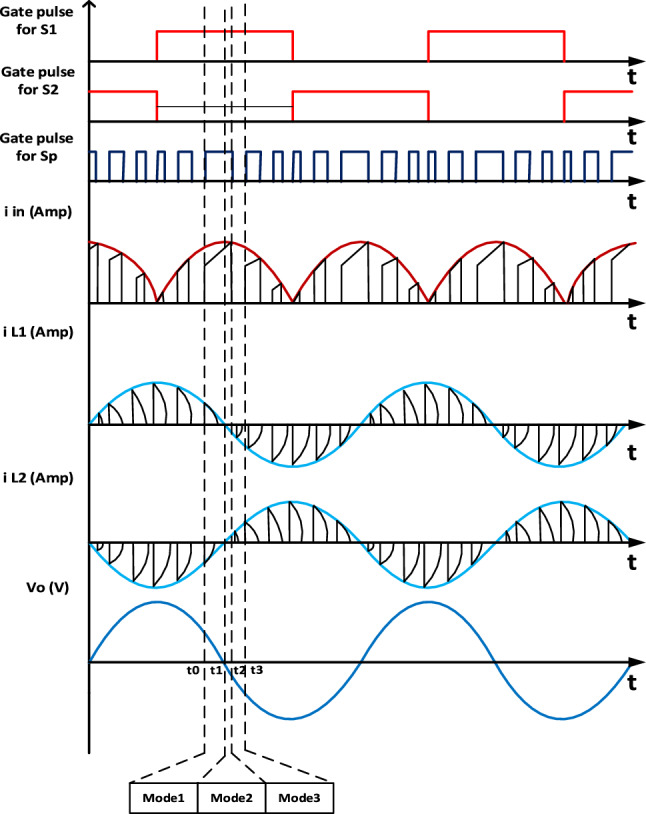
Analytical waveforms of the proposed topology.

In this mode switch ${S}_{{\varvec{p}}}$ and switch ${S}_{1}$ are ON, turning on switch ${S}_{{\varvec{p}}}$, inductor ${L}_{{\varvec{m}}}$ charged by source voltage (${V}_{{\varvec{i}}{\varvec{n}}}$) through switch ${S}_{{\varvec{p}}}$. Switch ${S}_{1}$ is ON and diode ${D}_{2}$ is forward biased. Switch ${S}_{2}$ is OFF and diode ${D}_{1}$ is reverse biased. Current in the mutual inductance ${L}_{{\varvec{m}}}$ rises linearly with slope of ${{\varvec{V}}}_{{\varvec{i}}{\varvec{n}}}$**/**${{\varvec{L}}}_{{\varvec{m}}}$. During this mode of operation, the capacitor ${C}_{0}$ is charged by the difference between the input voltage ${{\varvec{V}}}_{{\varvec{i}}{\varvec{n}}}$ and the first secondary voltage ${{\varvec{V}}}_{{\varvec{l}}1}$. This mode ends when Capacitor ${C}_{0}$ is fully charged and diode ${D}_{1}$ becomes forward biased. Figure 2a shows this mode of operation. Analytical waveforms of input current (${I}_{{\varvec{i}}{\varvec{n}}}$), current through each inductor (${I}_{{\varvec{L}}1}$ and ${I}_{{\varvec{L}}2}$), output voltage (${V}_{{\varvec{o}}}$) and gating pulses over a two switching cycles are demonstrated in Fig. 3.

### Mode 2 (${{\varvec{t}}}_{1},{{\varvec{t}}}_{2}$)

In this mode, diode ${D}_{1}$ becomes forward biased and diode ${D}_{2}$ becomes reverse biased. The adverse effects of leakage inductance will avoid using with the coupled inductor; consequently, reduce the stresses and voltage spikes on the switches. This mode ends when switch ${S}_{{\varvec{p}}}$ is turned OFF and Fig. 2b illustrates this mode of operation.

### Mode 3 (${{\varvec{t}}}_{2},{{\varvec{t}}}_{3}$)

During this mode, switch ${S}_{{\varvec{p}}}$ is turned OFF and switch ${S}_{1}$ still ON. The magnetizing inductor ${L}_{m}$ starts to discharge through inductor ${L}_{p}$. As a result of the coupling between ${L}_{p}$ and ${L}_{1}$, the power will transfer to load through ${L}_{1}$. Furthermore, the output capacitor ${C}_{{\varvec{o}}}$ start to discharge through load. The leakage energy associated with the leakage inductance ${L}_{1{\varvec{k}}}$ will be delivered to the load through the diode ${D}_{2}$. This mode ends when switch ${S}_{{\varvec{p}}}$ is turns ON again and Fig. 2c shows this mode of operation.

## Mathematical analysis

The proposed converter’s operation is symmetrical and the same in both half cycles of the output voltage waveform, so only one switching cycle of the negative half cycle of the output voltage is considered for analysis. Assumptions considered in the analysis include the following:

(i) Disregard all parasitic components, on-state resistances of the active switches, and forward voltage drops of the diodes.
(ii) Sole consideration of the leakage inductance of the coupled inductor in the voltage gain calculation, with neglect in other parts for the sake of simplifying calculations.
(iii) The capacitor ${C}_{{\varvec{o}}}$ is assumed to be sufficiently large, allowing it to be treated as a constant voltage source throughout a switching cycle.

### Voltage gain

Let Np, N1, N2 be the number of turns on primary, secondary 1 and secondary 2, and **n = (N1/Np) = (N2/Np)** for the topology shown in Fig. 1. Let ${L}_{{\varvec{P}}}$ be the primary inductance, there for L1 can be written as following^30^

$$L1=L2={n}^{2}*{L}_{p}$$

$$Lp={L}_{m}+{L}_{pk}$$

where ${L}_{{\varvec{P}}{\varvec{K}}}$ is the leakage inductance associated with primary winding, ${L}_{{\varvec{m}}}$ is the magnetizing inductance. Coefficient of coupling (k) is defined as following:

$$k=\frac{{L}_{{\varvec{m}}}}{{L}_{{\varvec{m}}}+{L}_{{\varvec{P}}{\varvec{K}}}}$$

**During switch on time**:- (${{\varvec{S}}}_{{\varvec{p}}}$ **is ON and** ${{\varvec{S}}}_{2}$ **is ON**)

Voltage across magnetizing inductance (${V}_{Lm}$) is given as

$${V}_{Lm}={k*V}_{in}$$

**During switch off time:- **(${{\varvec{S}}}_{{\varvec{p}}}$ **is OFF and** ${{\varvec{S}}}_{2}$ **is ON**).

The relation between current through the magnetizing inductance (${i}_{Lm}$) and current through the leakage inductance (${i}_{Lp}$) is given as follows

1 $${i}_{Lm}=\left(1+n\right){i}_{Lp}$$

the relation between output voltage (**Vo**) and voltages across inductors (${\mathbf{V}}_{\mathbf{L}\mathbf{p}}$ and ${\mathbf{V}}_{\mathbf{L}2}$) can be written as

2 $$-{V}_{o}={V}_{Lp}+{V}_{L2}$$

where

$$\begin{aligned} & V_{Lp} = V_{Lm} + V_{Lpk} \\ & V_{L2} = V_{L2k} + V_{Ln2 } = n^{2} V_{Lpk} + n V_{Lm } \\ \end{aligned}$$

Substituting $${V}_{Lp}$$, $${V}_{L2}$$ in (2)

3 $$\begin{aligned} & - V_{o} = V_{Lm } + V_{Lpk } + n^{2} V_{Lpk} + n V_{Lm } \\ & - V_{o} = \left( {1 + n^{2} } \right)V_{Lpk} + \left( {1 + n} \right) V_{Lm } \\ \end{aligned}$$

From (1), ${V}_{Lm}$ can be written as

$${V}_{Lm}={L}_{m}\frac{{di}_{Lm}}{dt}={L}_{m}\frac{{d\left(1+n\right)i}_{Lp}}{dt}=(1+n){L}_{m}\frac{{di}_{Lp}}{dt}$$

4 $$\frac{{{\varvec{d}}{\varvec{i}}}_{{\varvec{L}}{\varvec{p}}}}{{\varvec{d}}{\varvec{t}}}=\frac{{{\varvec{v}}}_{{\varvec{L}}{\varvec{m}}}}{{\left(1+{\varvec{n}}\right){\varvec{L}}}_{{\varvec{m}}}\boldsymbol{ }}$$

5 $${V}_{Lpk}={L}_{pk}\frac{{di}_{Lp}}{dt}={L}_{pk}\frac{{V}_{Lm}}{{\left(1+n\right)L}_{m}}$$

By compensate (4), (5) into (3):

$$\begin{aligned} & \left( {1 + n^{2} } \right)L_{pk} \frac{{v_{Lm} }}{{\left( {1 + n} \right)L_{m} }} + \left( {1 + n} \right)V_{Lm } = - V_{o} \\ & v_{Lm} \left( {\frac{{\left( {1 + n} \right)^{2} L_{m} + \left( {1 + n^{2} } \right)L_{pk} }}{{\left( {1 + n} \right)L_{m} }}} \right) = - V_{o} \\ \end{aligned}$$

From coefficient of coupling equation:

$${L}_{pk}={\frac{(1-k)}{k}L}_{m}$$

So,

6 $$\begin{aligned} & v_{Lm} \left( {\frac{{\left( {1 + n} \right)^{2} L_{m} + \left( {1 + n^{2} } \right)\frac{{\left( {1 - k} \right)}}{k}L_{m} }}{{\left( {1 + n} \right)L_{m} }}} \right) = - V_{o} \\ & v_{Lm} = \frac{{\left( {1 + n} \right)k}}{{\left( {n^{2} + 2nk + 1} \right)}}( - V_{o} ) \\ \end{aligned}$$

Applying volt–second balance across mutual inductance **(**${L}_{{\varvec{m}}}$**)** gives:

$${v}_{Lm}=k{v}_{in}d+\frac{\left({-v}_{o}\right)\left(1+n\right)k}{\left({n}^{2}+2nk+1\right)}\left(1-d\right)=0$$

where **d** is the duty cycle. Solving the above equation gives:

7 $$\frac{{v}_{o}}{{v}_{in}}=\left(\frac{d}{1-d}\right)\left(\frac{{n}^{2}+2nk+1}{1+n}\right)=M(d)$$

When **k = 1**, Eq. (7) becomes

$$\frac{{v}_{o}}{{v}_{in}}=\left(\frac{d}{1-d}\right)\left(\frac{{(1+n)}^{2}}{1+n}\right)=M(d)$$

8 $$\frac{{v}_{o}}{{v}_{in}}=\left(\frac{d}{1-d}\right)\left(1+n\right)=M(d)$$

From the above equation, in order to get output voltage (${v}_{o}$) of frequency ω radians per second from input voltage (${v}_{in}$), duty cycle can be varied as per the following equation:

9 $$d\left(t\right)=\left(\frac{{v}_{o}\text{sin}\left(wt\right)}{\left(1+n\right){v}_{in}+{v}_{o}\text{sin}\left(wt\right)}\right)$$

The voltage boost value depends on the duty cycle (d) and the chosen for coupled inductor transformation ratio value. The switch S_p_ works with a sinusoidal pulse width modulation (SPWM) strategy, while S_1_ and S_2_ are working with a duty cycle of 50%. The transformer ratio for five times voltage boost is 4.

### Voltage stress across the switches:

#### For switch ${{\varvec{S}}}_{{\varvec{p}}}$

The maximum voltage stress across the active switch (${S}_{{\varvec{p}}}$) occurs during mode 3, i.e., when the switch (${S}_{{\varvec{p}}}$) turns off. The switch (${S}_{{\varvec{p}}}$) clamps between input and output terminals. Hence the maximum voltage stress across the active switch (${S}_{{\varvec{p}}}$) is

$${\text{v}}_{\text{sp}(\text{Max})}={\text{v}}_{\text{in}}+{\text{v}}_{\text{o}(\text{Max})}$$

#### For switches ${{\varvec{S}}}_{1}$ and ${{\varvec{S}}}_{2}$

The maximum voltage stresses across the active switches (${S}_{1}$ and ${S}_{2}$) are same and occur at peak value of the output voltage. Therefore, the maximum stress across these switches is ${\mathbf{v}}_{\mathbf{o}(\mathbf{M}\mathbf{a}\mathbf{x})}$.

## Design of the components

The design of the proposed topology for continuous conduction mode (CCM) operation requires determination of the values of the coupled inductor and output capacitor (Co).

### Design of coupled inductor

Let $\Delta {I}_{Lp}$ be the allowable current rise from normal current through inductor ${L}_{p}$.

**Figure 4:**
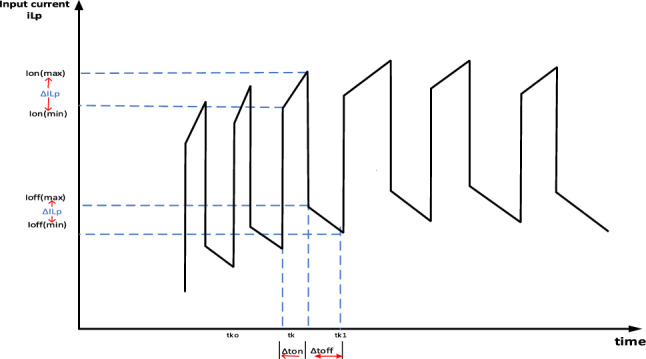
Section of primary inductor (${L}_{P}$) current waveform (sinewave track) where: ${I}_{on\left(max\right)}={I}_{{Lp}_{ref }}{\text{sin}}^{2}(wt)+\Delta {I}_{Lp}$; ${I}_{on\left(min\right)}={I}_{{Lp}_{ref }}{\text{sin}}^{2}\left(wt\right)-\Delta {I}_{Lp}$; ${I}_{off\left(max\right)}=\left({I}_{{Lp}_{ref }}{\text{sin}}^{2}\left(wt\right)+\Delta {I}_{Lp}\right)/\left(1+n\right)$; ${I}_{off\left(min\right)}=\left({I}_{{Lp}_{ref }}{\text{sin}}^{2}\left(wt\right)-\Delta {I}_{Lp}\right)/\left(1+n\right)$.

The voltage across inductor ${L}_{p}$ during $\Delta {\mathbf{t}}_{\mathbf{o}\mathbf{n}}$ as shown in Fig. 4 will be:-

$${V}_{in}={L}_{p}\Delta I/\Delta T$$

$$\frac{{v}_{in}}{{L}_{p}}=\left(\frac{{I}_{{Lp}_{ref }}{\text{sin}}^{2}(w{t}_{ko})+\Delta {I}_{Lp}}{{t}_{k}-{t}_{{k}_{0}}}\right) -\left(\frac{{I}_{{Lp}_{ref }}{\text{sin}}^{2}\left(w{t}_{ko}\right)-\Delta {I}_{Lp}}{{t}_{k}-{t}_{{k}_{0}}}\right)$$

$$\frac{{v}_{in}}{{L}_{p}}=\left(\frac{{I}_{{Lp}_{ref }}{(\text{sin}}^{2}\left(w{t}_{ko}\right){-\text{sin}}^{2}\left(w{t}_{ko}\right))+2*\Delta {I}_{Lp}}{{t}_{k}-{t}_{{k}_{0}}}\right)$$

Assume that

$$t_{k} - t_{{k_{0} }} = \Delta t_{on} ;\quad \sin w(t_{k} - t_{{k_{0} }} ) = w*\Delta t_{on} ;\quad t_{k} + t_{{k_{0} }} = 2t_{k}$$

So;

$$\frac{{v}_{in}}{{L}_{p}}=\left(\frac{{-I}_{{Lp}_{ref }}\text{sin}(2w{t}_{k})*w*\Delta {t}_{on}+2*\Delta {I}_{Lp}}{\Delta {t}_{on}}\right)$$

10 $$\Delta {t}_{on}=\frac{2*\Delta {I}_{Lp}*{L}_{p}}{{v}_{in}+{I}_{{Lp}_{ref }}*{L}_{p}*\text{sin}(2w{t}_{k})*w}$$

Similarly, $\Delta {\mathbf{t}}_{\mathbf{o}\mathbf{f}\mathbf{f}}$ can be calculated as

11 $$\Delta {t}_{off}=\frac{2*\Delta {I}_{Lp}*(1+n)*{L}_{p}}{{v}_{o}+{I}_{{Lp}_{ref }}*(1+n)*{L}_{p}*\text{sin}(2w{t}_{k})*w}$$

Total switching period (${\mathbf{T}}_{\mathbf{s}}$) will be

$${T}_{s}=\Delta {t}_{on}+\Delta {t}_{off}$$

$${T}_{s}=\frac{2*\Delta {I}_{Lp}*{L}_{p}}{{v}_{in}+{I}_{{Lp}_{ref }}*{L}_{p}*\text{sin}(2w{t}_{k})*w}+\frac{2*\Delta {I}_{Lp}*(1+n)*{L}_{p}}{{v}_{o}+{I}_{{Lp}_{ref }}*(1+n)*{L}_{p}*\mathit{sin}(2w{t}_{k})*w}$$

At the peak of the output voltage where $\mathbf{sin}(2\mathbf{w}{\mathbf{t}}_{\mathbf{k}})\cong 0$ and $\mathbf{sin}(\mathbf{w}{\mathbf{t}}_{\mathbf{k}})\cong 1$;

12 $${T}_{s}=\frac{2*\Delta {I}_{Lp}*{L}_{p}}{{v}_{in}}+\frac{2*\Delta {I}_{Lp}*(1+n)*{L}_{p}}{{v}_{o(max)}}$$

By simplifying Eq. (12);

13 $$\begin{aligned} & T_{s} = L_{p} \left[ {\frac{{2*\Delta I_{Lp} *\left( {v_{{o\left( {max} \right)}} + \left( {1 + n} \right)v_{in} } \right)}}{{v_{in} *v_{{o\left( {max} \right)}} }}} \right] \\ & L_{p} = \frac{{T_{s} *\left( {v_{in} *v_{{o\left( {max} \right)}} } \right)}}{{2*\Delta I_{Lp} *\left( {v_{{o\left( {max} \right)}} + \left( {1 + n} \right)v_{in} } \right)}} \\ \end{aligned}$$

After calculating ${\mathbf{L}}_{\mathbf{p}}$;${\mathbf{L}}_{1}$& ${\mathbf{L}}_{2}$ can be expressed as following:-

14 $${L}_{1}= {n}^{2}*{L}_{p}$$

15 $${L}_{2}= {n}^{2}*{L}_{p}$$

### Design of capacitor (${\mathbf{C}}_{\mathbf{o}}$)

The value of the output capacitance (${\text{C}}_{\mathbf{o}}$) depends on the maximum energy that can be transferred through the coupled inductor.

Assuming unity power factor, maximum energy is transferred from input to output at the peak of the output voltage. By equaling the increase in energy of output capacitor with the decrease in energy through coupled inductor; the result will be:-

16 $$\begin{aligned} & \frac{1}{2}L_{p} \frac{{\left( {1 + n^{2} + 2n} \right)}}{1 + n}\left[ {\left( {I_{{Lp_{ref } \left( {max} \right)}} + \Delta I_{Lp} } \right)^{2} - \left( {I_{{Lp_{ref } \left( {max} \right)}} - \Delta I_{Lp} } \right)^{2} } \right] \\ & \quad = \frac{1}{2}C_{o} \left[ {\left( {V_{{o\left( {{\text{max}}} \right)}} + \Delta V} \right)^{2} - \left( {V_{{o\left( {{\text{max}}} \right)}} - \Delta V} \right)^{2} } \right] \\ \end{aligned}$$

By simplifying Eq. (16);

17 $${C}_{o}=\frac{{L}_{p}*\left(1+n\right)*{I}_{{Lp}_{ref }(max)}*\Delta {I}_{Lp}}{{V}_{o(\text{max})}*\Delta V}$$

where ${\mathbf{I}}_{{\mathbf{L}\mathbf{p}}_{\mathbf{r}\mathbf{e}\mathbf{f}}(\mathbf{m}\mathbf{a}\mathbf{x})}$ is the maximum amplitude of the reference primary inductor current, and $\Delta \mathbf{V}$ is the maximum allowable ripple voltage across output capacitor.

### Efficiency calculation

Power losses of any semiconductor switch, IGBT or diode, can be divided into conduction losses, switching losses, and blocking losses. The blocking losses are low compared to the other two losses and can be neglected^31^.

The power losses in the coupled inductors (${L}_{{\varvec{p}}}, {L}_{1} and {L}_{2}$), and the main capacitor (${C}_{{\varvec{o}}}$) can be calculated with regular topology in any electric circuit. Then total power loss is the sum of losses in switches and all losses in electric passive elements:

18 $$\upeta = \frac{{P_{o} }}{{P_{in} }} = \frac{{P_{L} }}{{P_{in} }} = \frac{{P_{L} }}{{P_{L} + \sum P_{Losses} }}$$

Consequently, the proposed converter efficiency depends on the switch’s losses as well as the passive electric element design. Therefore, efficiency varies from 60 to 90% according to passive electric element value.

### Control technique

Sinusoidal Pulse Width Modulation (SPWM) is a commonly used control strategy for inverters in power electronics. SPWM is employed to generate a sinusoidal waveform by modulating the width of the pulses in a pulse train.

**Figure 5:**
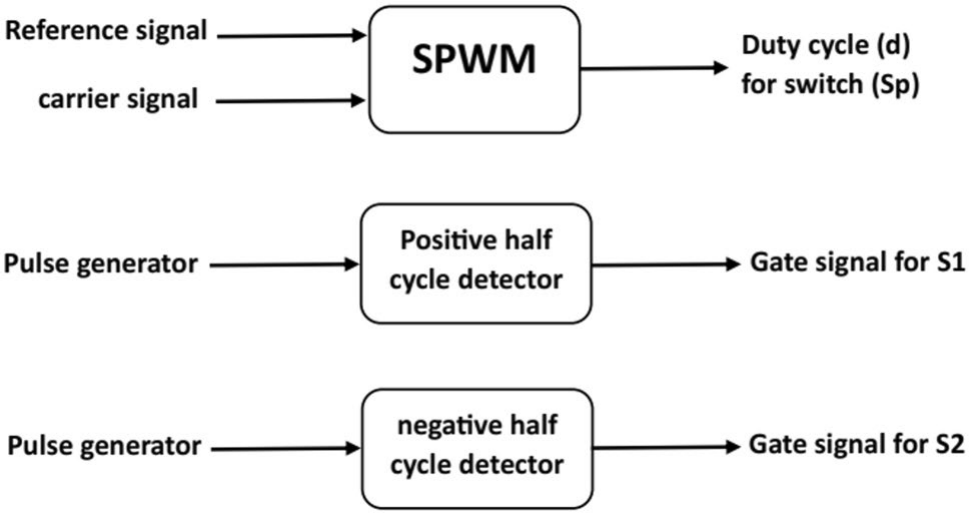
Control block diagram of the proposed converter.

In this paper, A SPWM and ON–OFF control techniques are used for controlling switches ${S}_{{\varvec{p}}}$, ${S}_{1}$ and ${S}_{2}$. Control block diagram of the proposed converter is shown in Fig. 5.

**Figure 6:**
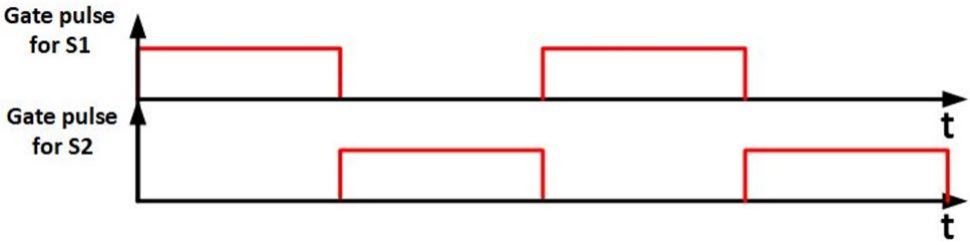
Gate pulses for ${S}_{1}$ and ${S}_{2}$*.*

**Figure 7:**
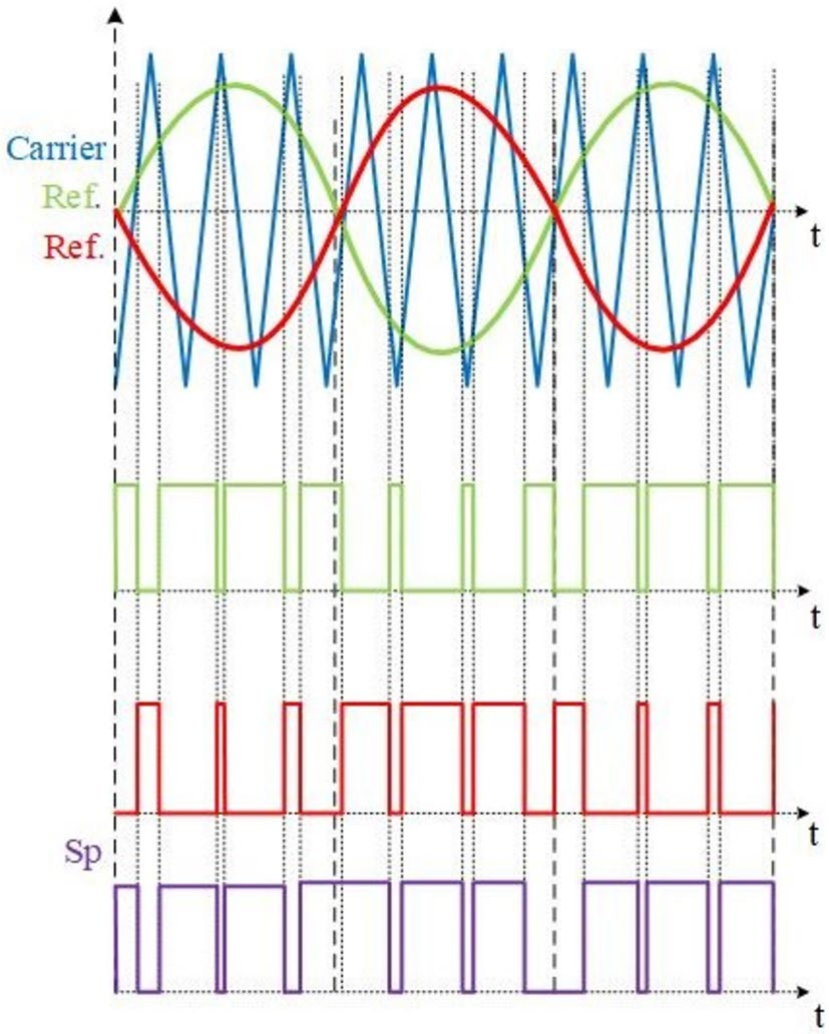
SPWM for $${S}_{p}$$.

Switches ${S}_{1}$ and ${S}_{2}$ are controlled using ON–OFF regulation, illustrated in Fig. 6. Meanwhile, Fig. 7 depicts the implementation of the SPWM technique employed to drive switch ${S}_{{\varvec{p}}}$.

In both simulation and experimental conditions, switch ${S}_{{\varvec{p}}}$ are driven by SPWM signal with switching frequency of 5 KHZ. Also, switches ${S}_{1}$ and ${S}_{2}$ are driven by ON–OFF control with duty of 50%.

## Simulation study

**Figure 8:**
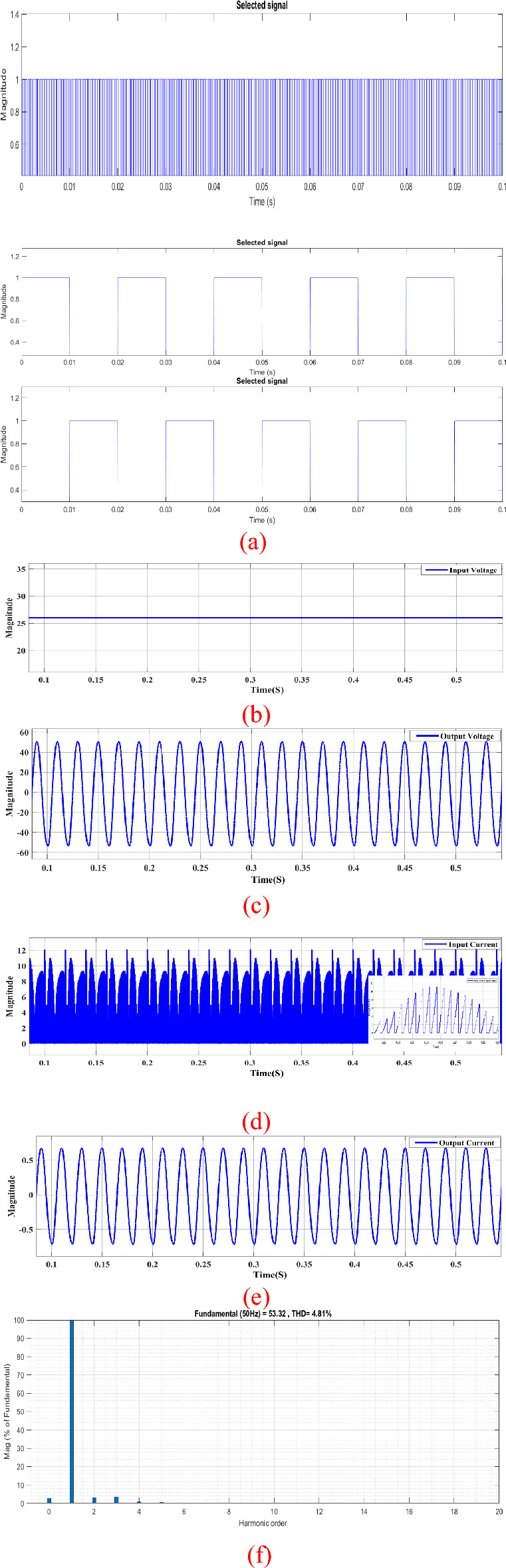
Steady-state simulation results of proposed topology for resistive load. (**a**) Pulses for active switches (${S}_{p}$_,_ *S*_1,_ *S*_2_). (**b**) Input DC source. (**c**) Output AC source. (**d**) Input DC current. (**e**) Output AC current. (**f**) THD.

The power circuits shown in Fig. 1 with the control system illustrated in Fig. 5 are built using **MATLAB/SIMULINK.** Performance of the proposed circuit is analyzed at different frequencies and different duty cycles to study the circuit performance at different operation conditions. Figures 8 and 10 shows the circuit behavior at boost mood and Fig. 11 shows the circuit behavior at buck mood. The system parameters used at one operating condition are as shown in Table 1.

**Table 1:** Simulation parameter.

Parameters			
${\text{L}}_{\text{m}}$	5 mH	K	0.5
${\text{L}}_{p}$	5 mH	${\text{C}}_{\text{O}}$	220 μF
${\text{L}}_{1}$	0.5 mH	${\text{R}}_{\text{O}}$	70 Ω
${\text{L}}_{2}$	0.5 mH	${\text{F}}_{\text{O}}$	50 Hz
F(S_P_)	5000 Hz		

The input voltage was about 26V DC as in Fig. 8b and input current reaches 10 A peak as Fig. 8d. As the proposed topology works in boost mood, it will exhibit a voltage gain approximately twice the DC input voltage about 50 V AC peak as shown in Fig. 8c. The output current was about 0.75A peak as shown in Fig. 8e. Additionally, the output voltage and current follow sinusoidal waveforms, as illustrated in Fig. 8c,e.

The Total Harmonic Distortion (THD) of the output voltage is approximately 4.81%, as depicted in Fig. 8f. The input current features chopped DC components, occurring when the switch ${S}_{{\varvec{p}}}$ is fired at a switching frequency of 5 kHz, as illustrated in Fig. 8d.

**Figure 9:**
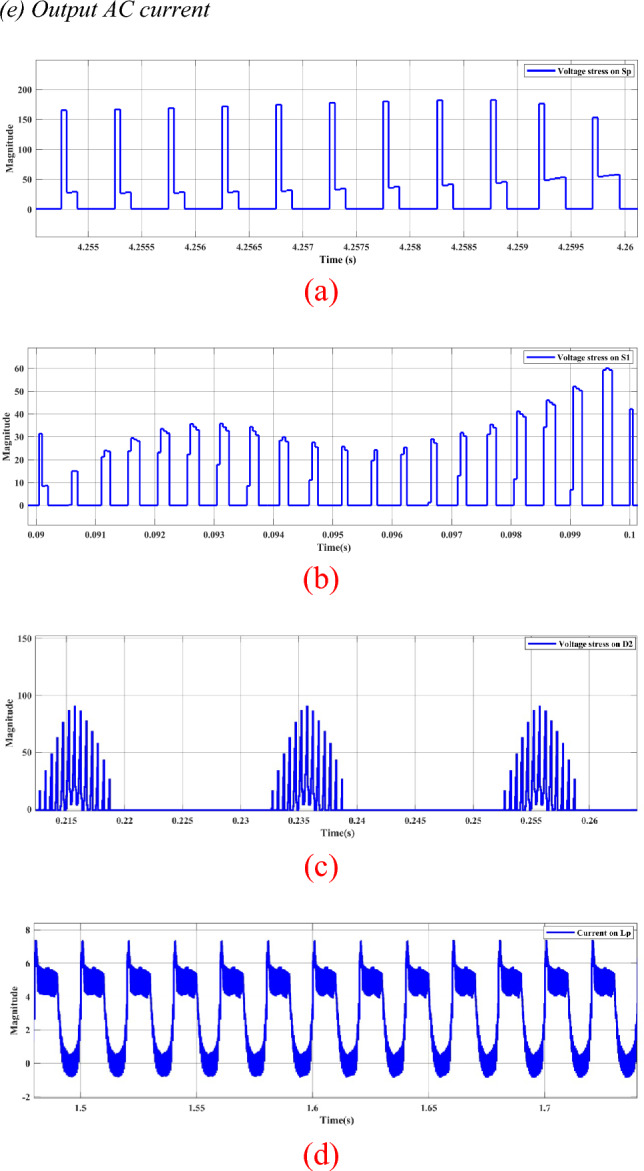
Steady-state simulation results for voltages stress and current of switches, diodes and inductor. (**a**) Voltage stress for* S*_*p*_. (**b**) voltage stress on* S*_1_. (**c**) Voltage stress on* D*_2_. (**d**) Current through *L*_*p*_*.*

The switch ${S}_{{\varvec{p}}}$ is triggered using Sinusoidal Pulse Width Modulation (SPWM), as depicted in Fig. 8a with a switching frequency of 5 kHz. On the other hand, switches ${S}_{1}$ and ${S}_{2}$ are activated through an ON–OFF controller with a frequency of 50 Hz and a duty cycle of 0.5. This setup ensures a symmetric waveform, where switch ${S}_{1}$ operates during the positive half cycle, and switch ${S}_{2}$ operates during the negative half cycle. The voltages stress and current of switches, diodes and inductor are depicted in Fig. 9.

**Figure 10:**
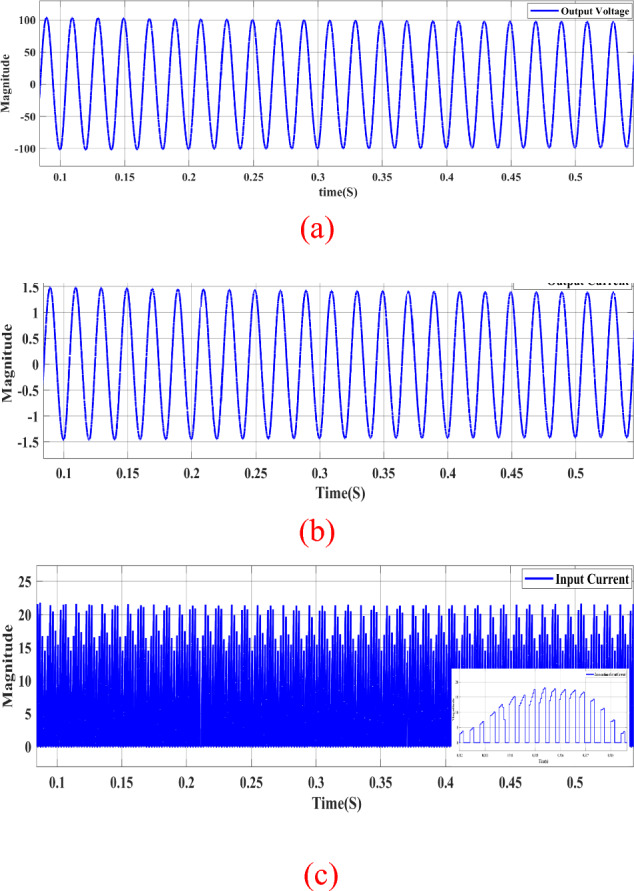
Steady-state simulation results of proposed topology for resistive load for1KHZ of ${S}_{{\varvec{p}}}$. (**a**) Output AC voltage. (**b**) Output AC current. (**c**) Input DC current.

In Fig. 10a, the output voltage is presented at a switching frequency of 1 kHz for switch ${S}_{{\varvec{p}}}$, revealing a boosting gain of nearly 4. Figure 10b displays the output current at the same switching frequency of 1 kHz, showcasing sinusoidal waveforms for both the output voltage and current.

Simultaneously, in Fig. 10c, the input current exhibits chopped DC components, occurring as switch ${S}_{{\varvec{p}}}$ is fired with a switching frequency of 1 kHz. The Total Harmonic Distortion (THD) of the output voltage at this switching frequency is approximately 9.36%, as indicated in the data.

**Figure 11:**
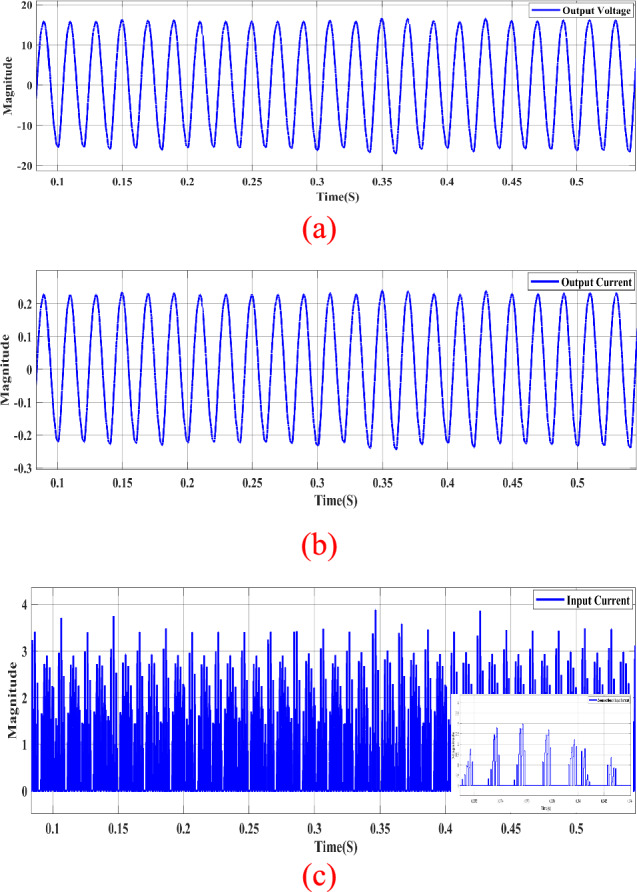
Steady-state simulation results of proposed topology for resistive load for 50KHZ of ${S}_{{\varvec{p}}}$. (**a**) Output AC voltage. (**b**) Output AC current. (**c**) Input DC current.

Figure 11 demonstrates the circuit operating in buck mode. By modifying the signal of switch ${S}_{{\varvec{p}}}$, the circuit transitions to buck mode, as illustrated in Fig. 10. The output voltage in Fig. 10a indicates that the circuit is in buck conditions, displaying a voltage reduction of nearly half the DC input voltage.

Moreover, both the output voltage and current exhibit sinusoidal waveforms, as depicted in Fig. 11a,b.

The Total Harmonic Distortion (THD) of the output voltage is approximately 5.72%, as mentioned in the description. Simultaneously, the input current in Fig. 11c displays chopped DC components due to the firing of switch ${S}_{{\varvec{p}}}$ with a switching frequency of 50 kHz.

## Experiential study

A prototype Experimental system is setup in the laboratory with parameter shown in Table 2.

**Table 2:** Experimental parameter.

Parameters			
${\text{L}}_{\text{m}}$	5 mH	K	0.5
${\text{L}}_{p}$	5 mH	${\text{C}}_{\text{O}}$	200 μF
${\text{L}}_{1}$	0.5 mH	${\text{R}}_{\text{O}}$	70 Ω
${\text{L}}_{2}$	0.5 mH	${\text{F}}_{\text{O}}$	50 Hz
F(S_P_)[Boost]	5000 Hz	F(S_P_)[Buck]	50 kHz

**Figure 12:**
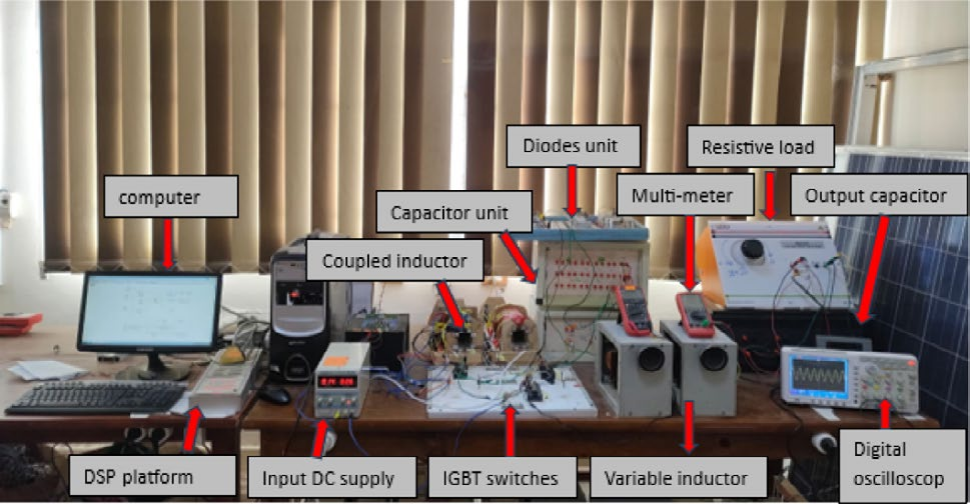
Experimental setup.

A 1200 V, 150 A dual IGBT module.

(CM100DY-24H NO. A10H86) is used for implementing switches (${S}_{{\varvec{p}}}$, ${S}_{1}$ and ${S}_{2}$). Diodes (D1 and D2) are implemented using power diodes.

(IXYSDSDI35-10A). Digital signal processor controller is used to give corresponding switching pulses to all active switches. Developed experimental setup is shown in Fig. 12.

**Figure 13:**
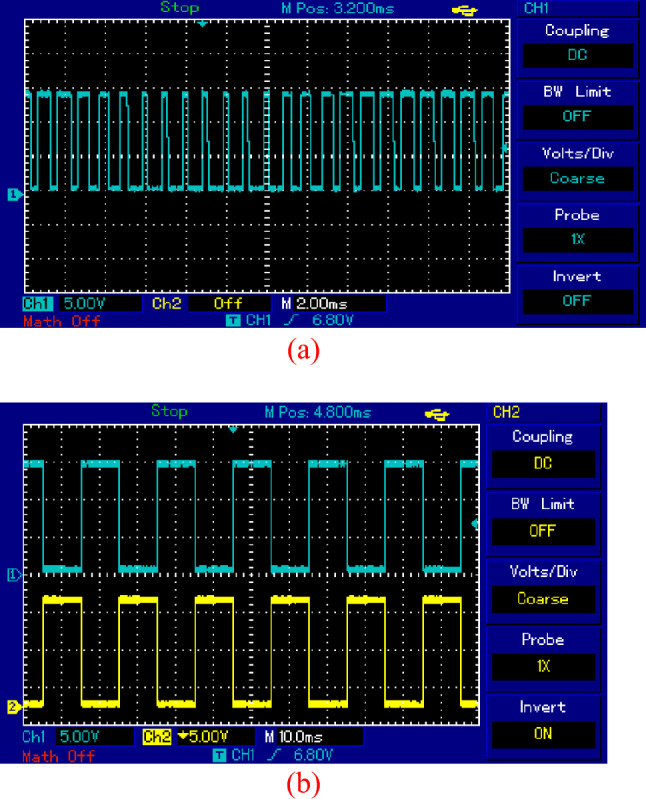
Pulses for active switches. (**a**) SPWM for *S*_*p*_. (**b**) pulses for *S*_1_ and* S*_2_*.*

The pulses used for trigger switches Sp, S1 and S2 is shown in Fig. 13. Figure 13a shows SPWM foe switch Sp at 5KHZ while Fig. 13b shows the pulses for S1 and S2 at frequency of 50HZ.

**Figure 14:**
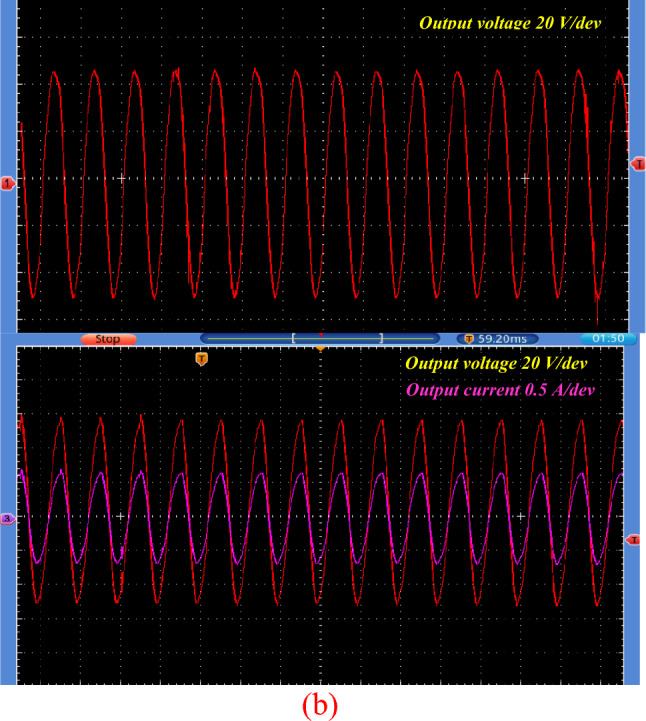
Steady-state experimental results of proposed topology for resistive load for 5KHZ of *S*_*p*_. (**a**) Output AC voltage. (**b**) Output AC current and voltage.

The input voltage was about 26V DC and input current reaches 3.5 A. The circuit operates in a buck–boost configuration. When the proposed topology works in boost mood, it will exhibit a voltage gain approximately twice the DC input voltage about 52V AC peak (32.4 V rms) as shown in Fig. 14a. The output current was about 0.75A peak (0.43 A rms) as shown in Fig. 14b.

**Figure 15:**
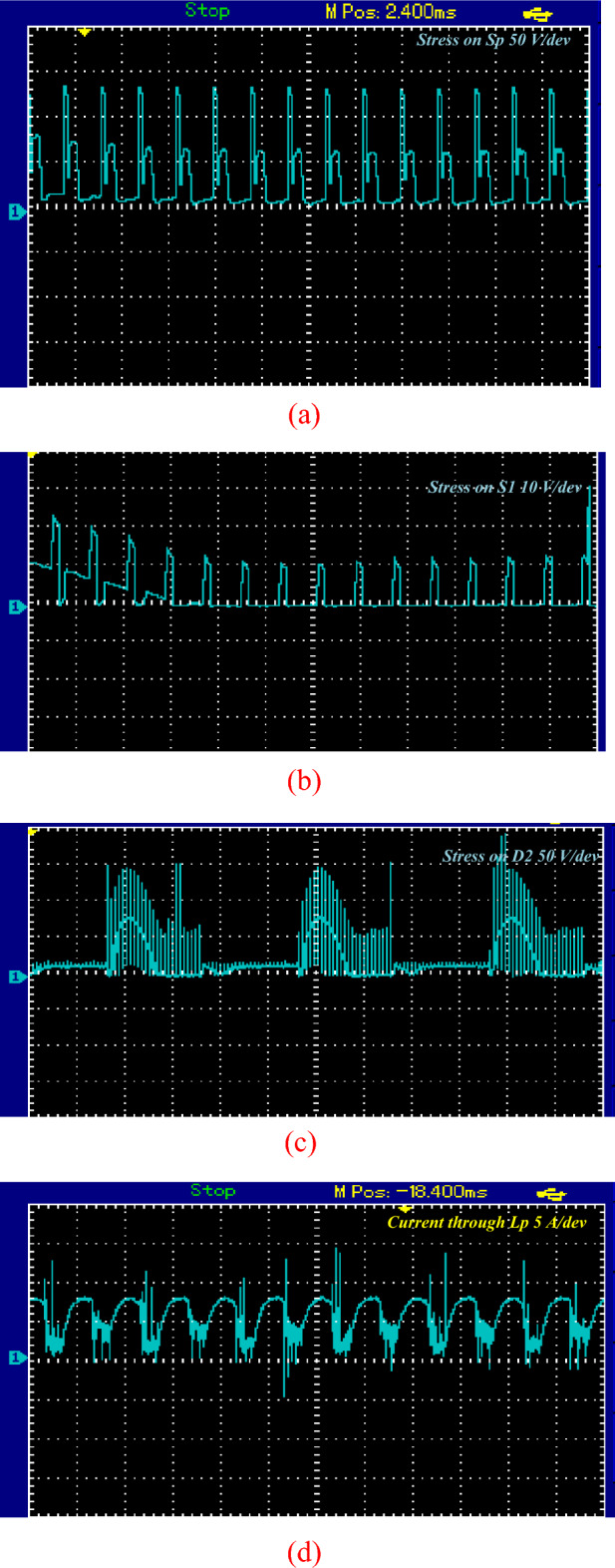
Steady-state experimental results for voltages stress and current of switches, diodes and inductor. (**a**) Voltage stress for* S*_*p*_. (**b**) voltage stress on* S*_1_. (**c**) Voltage stress on* D*_2_. (**d**) Current through *L*_*p*_*.*

**Figure 16:**
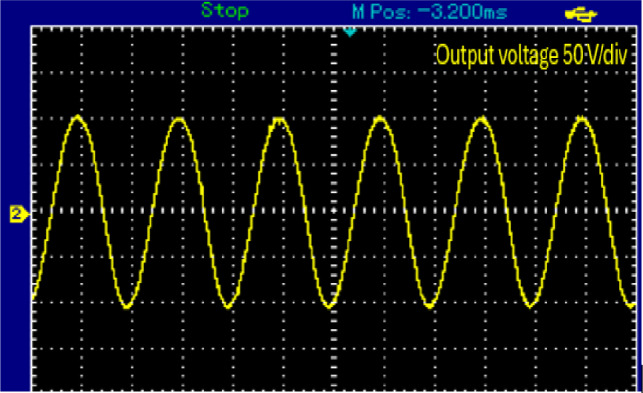
Steady-state experimental results of proposed topology for resistive load for1KHZ of ${S}_{{\varvec{p}}}$.

Figure 15 displays the experimental results depicting the voltage stress across S_p_, S_1_, and D_1_, as well as the current through L_p_. The data demonstrates a high degree of agreement between experimental findings and simulation results. Figure 16 shows the circuit behavior in boost mode when working at switching of 1 kHz.

**Figure 17:**
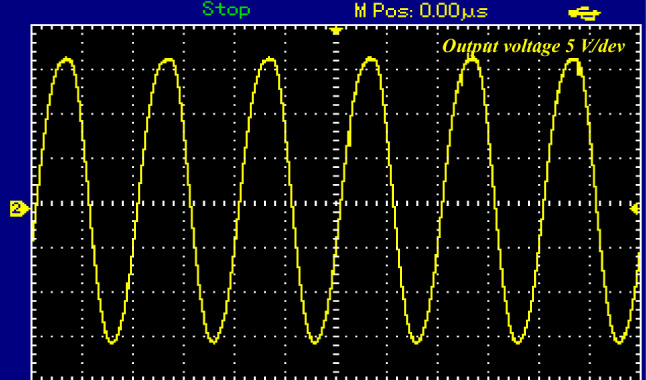
Steady-state experimental output voltage of proposed topology at buck mood for resistive load.

When the proposed topology works in buck mood, it will exhibit a voltage reduction of approximately half the DC input voltage about 16 V AC peak as shown in Fig. 17.

**Figure 18:**
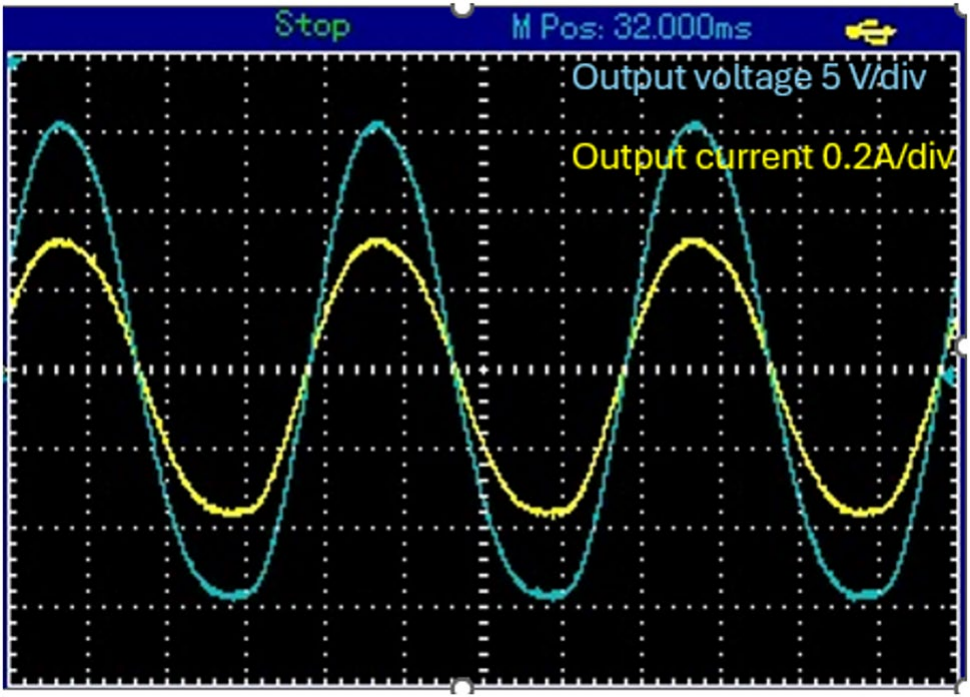
Steady-state experimental output voltage and current proposed topology at buck mood for resistive load.

Figure 18 shows the output voltage and current in this mode. Both the output voltage and current manifest as sinusoidal waveforms.

The experimental results closely align with both simulation and analytical findings, thereby validating the proposed scheme.

**Figure 19:**
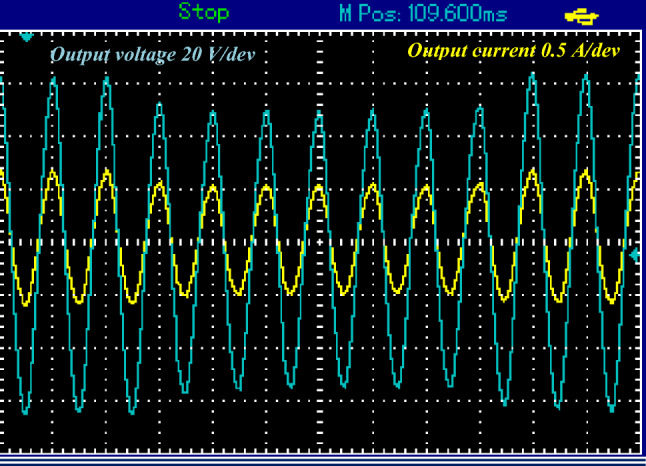
Converter response during step change in load.

**Figure 20:**
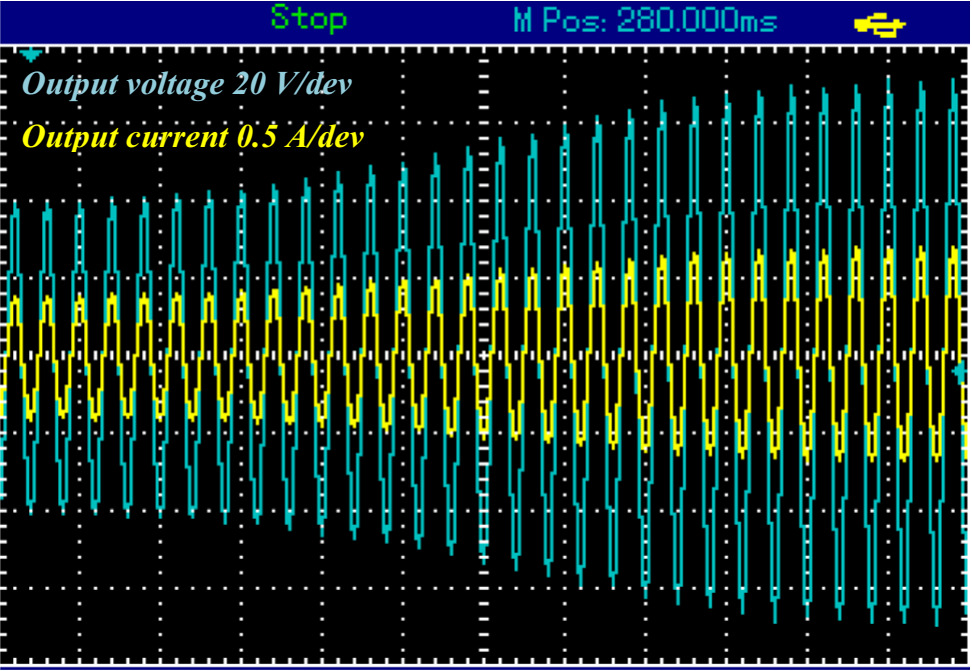
Converter response during input voltage variation.

Figure 19 illustrates the response of the output voltage and current when the load changes from 56 to 70 Ω for 0.14 s and then returns to 56 Ω. This case represents a 25% change in the rated load, the converter output is sinusoidal wave form. Figure 20 shows the response of the output voltage and current when the input voltage changes gradually from 23 to 37 V in 0.2 s. It is clear that the waveforms are appropriate in response to changes in load and input voltage, maintaining the desired performance and stability.

## Conclusion

In this paper, a single stage high gain DC–AC converter based on coupled inductors with reduce number of semiconductor switches has been presented. It provides many merits when it is compared with other DC–AC converters such as: higher voltage gains (AC output voltage changes and can reach nearly 5 times of input DC voltage), lower cost, simpler control system, lower switching loss, and low number of switches are used (only three switches are used). The operation, analysis and complete design of the proposed topology are introduced. A simulation study has been performed to check the system performance where the output current and voltage with resistive load is nearly sinusoidal with THD less than 10%. And finally, an experimental setup has been implemented a good agreement between simulation and experimental results.

## Data Availability

The datasets used and/or analyzed during the current study available from the corresponding author on reasonable request.
